# Roles of MicroRNA-21 in Skin Wound Healing: A Comprehensive Review

**DOI:** 10.3389/fphar.2022.828627

**Published:** 2022-02-28

**Authors:** Jie Xie, Weizhou Wu, Liying Zheng, Xuesong Lin, Yuncheng Tai, Yajie Wang, Le Wang

**Affiliations:** ^1^ Department of Emergency Medicine, Taizhou Central Hospital (Taizhou University Hospital), Taizhou, China; ^2^ Department of Urology, Maoming People’s Hospital, Guangdong, China; ^3^ Postgraduate Pepartment, First Affiliated Hospital of Gannan Medical College, Ganzhou, China; ^4^ Department of Burn Surgery, Taizhou Central Hospital (Taizhou University Hospital), Taizhou, China

**Keywords:** MicroRNA-21, wound healing, target, function, mechanism

## Abstract

MicroRNA-21 (miR-21), one of the early mammalian miRNAs identified, has been detected to be upregulated in multiple biological processes. Increasing evidence has demonstrated the potential values of miR-21 in cutaneous damage and skin wound healing, but lack of a review article to summarize the current evidence on this issue. Based on this review, relevant studies demonstrated that miR-21 played an essential role in wound healing by constituting a complex network with its targeted genes (i.e., PTEN, RECK. SPRY1/2, NF-κB, and TIMP3) and the cascaded signaling pathways (i.e., MAPK/ERK, PI3K/Akt, Wnt/β-catenin/MMP-7, and TGF-β/Smad7-Smad2/3). The treatment effectiveness developed by miR-21 might be associated with the promotion of the fibroblast differentiation, the improvement of angiogenesis, anti-inflammatory, enhancement of the collagen synthesis, and the re-epithelialization of the wound. Currently, miRNA nanocarrier systems have been developed, supporting the feasibility clinical feasibility of such miR-21-based therapy. After further investigations, miR-21 may serve as a potential therapeutic target for wound healing.

## Introduction

Skin wound healing is an essential physiological process to maintain the integrity of the skin (Cui et al., 2020). It is a complex dynamic and highly orchestrated process and involves coordinated interactions among cells, growth factors, and extracellular matrix (Liao et al., 2018). Wound healing can be typically subdivided into three main phases: the inflammatory phase, a proliferative phase, and a remodeling phase (Wada et al., 2021). At the inflammatory phase of wound healing, platelets released factors, such as chemokines and growth factors, attract neutrophils and macrophages infiltration for bacterial eradication and debridement (Li et al., 2021). Then various bioactive substances including epidermal growth factor (EGF), platelet-derived growth factor (PDGF), and transforming growth factor *α* (TGF-α) stimulate fibroblast proliferation and angiogenesis, thereby promoting the formation of granulation tissue (Lee et al., 2020).

Despite advances in the field of wound healing, there is still a significant unmet need for therapeutics to promote wound healing. With the aging population and the rise in the incidence of diabetic foot ulcers, pressure ulcers, and venous leg ulcers, nonhealing wounds can not only significantly reduce the quality of patients’ life but also bring huge economic losses to society (Ross 2021). Treating wounds and associated comorbidities is estimated to spend 5.3 billion annually in the United Kingdom and $25 billion in the United States (Guest et al., 2015; Sen et al., 2009). So far, there are only four treatment modalities approved by the Food and Drug Administration for treating chronic cutaneous wounds (Hamdan et al., 2017). These include a bioengineered human skin equivalent, a recombinant human platelet-derived growth factor, and two dermal substitutes (Naskar and Kim 2020). Furthermore, the estimated numbers of diabetics worldwide are as high as 630 million by 2045. Therefore, further studies of the underlying mechanism of wound healing are thus needed so that new therapeutic targets can be developed.

miRNAs are short, endogenous, non-coding RNA molecules, consisting of approximately 20–22 nucleotides (Tan et al., 2020). miRNAs are able to bind the 3′ untranslated region (UTR) of the target gene mRNA to promote the degradation of mRNA or induce translational repression, thus realizing the post-transcriptional regulation of gene expression (Wang et al., 2019). Recently, several studies have found that microRNAs (miRNAs) participated in the regulation of many biological processes, including differentiation, proliferation, apoptosis, or cell migration. Thus the dysregulation of miRNAs has been frequently observed in many pathologies, including wound healing and they have been proposed as therapeutic targets for many diseases. Wang et al. (Wang et al., 2018) reported that miR-129 or -335 overexpression promotes wound healing by inhibiting MMP-9 protein expression through targeting Sp1. Chen et al. (Chen et al., 2020) found that miR-139-5p expression was down-regulated by porcine acellular dermal matrix (ADM) and enhanced cutaneous wound healing by inhibiting the expression of JAG1 and Notch1. A recent study showed that miR-21 was also involved in wound healing (Simoes et al., 2019). miR-21 is aberrantly expressed in most human tumors and is one of the most investigated miRNAs (Ambros 2003). Moreover, wound healing shares similar molecular mechanisms with tumorigenesis (Werner and Grose 2003). In recent years, the role of miR-21 in wound healing has attracted the increasing attention from researchers. In the present article, we summarize the current knowledge about miR-21 in wound healing.

## Overview of miR-21

MicroRNA-21 (miR-21), a gene located on chromosome 17 of *Homo sapiens* (17q.23.1), was one of the early mammalian miRNAs identified. miR-21 can be found in the cytosol, extracellular exosome, and multiple organs (Varikuti et al., 2021). In line with other miRNAs, miR-21 functionally regulates its targeting mRNA through interaction with the 3 untranslated regions (UTR), forming the RNA-induced silencing complex for the targeted gene silencing. As predicted, about 170 genes are under the regulation of miR-21, but the biological functions of the miR-21-targeted gene complexes were only experimentally validated in a small number (Kertesz et al., 2007). Commonly, miR-21 is upregulated in multiple biological processes, including inflammatory, cancer, and fibrosis. miR-21 expression levels in serum or sputum can be applied as a diagnostic biomarker of various diseases. The functions of miR-21 in these diseases are strongly associated with the binding to the non-coding region of the target gene at the post-transcriptional level. As reported, miR-21 may regulate multiple target genes, including phosphatase and Tensin Homolog (PTEN), Transcription Factor Dp Family Member 3 (TFDP3), HMG-Box Transcription Factor 1 (HMG-Box Transcription Factor 1), Fatty Acid Binding Protein 7 (Fatty Acid Binding Protein 7), Hypoxia-inducible factor 1-alpha (HIF-α), Programmed Cell Death 4 (PDCD4), Transforming growth factor-beta (TGF-β), SMAD Family Member 7 (SMAD), Interleukin 12 (IL-12), and Tissue inhibitors of metalloproteinases 3 (TIMP-3), etc (Zhang T et al., 2020). Increasing evidence has demonstrated the potential value of miR-21 in organ injuries, i.e., cerebral injury (Yan et al., 2021), myocardial ischemia/reperfusion injury (Yuan and Fu 2021), and liver injury (ShamsEldeen et al., 2021). Besides, numerous studies have also identified the critical roles of miR-21 in cutaneous damage and wound healing (Abdel-Gawad et al., 2021; Ma et al., 2020). Due to the lack of review articles that focus on the association between miR-21 expression and wound healing, thus it is worth summarizing all the current evidence on this issue.

### The Roles of miR-21 in Skin Wound Healing miR-21 Promotes Wound Healing by Down-Regulating PTEN and RECK in Protein Level and Activating MAPK/ERK Signaling Cascade

Reepithelialization is a critical part of wound healing (Park et al., 2018). It has been reported that keratinocytes-derived signals played an important role for dermal fibroblasts to form the functional epidermal (Ezhilarasu et al., 2019). In addition, keratinocytes-fibroblasts interaction induces collagen synthesis and contraction (Schafer et al., 1989). Microvesicles (MVs) have a diameter of 100–1,000 nm and are actively generated by a variety of cells, including epithelial cells (Casado et al., 2017). They mediate cell-to-cell communication by transferring microRNAs, chemokines, and cell surface receptors from origin-cells to target-cells (Stahl et al., 2015). It has been reported that MVs derived from keratinocytes promoted fibroblast cell migration by activating ERK1/2, Smad, and p38 signaling pathways (Bi et al., 2016; Huang et al., 2015). Furthermore, miR-21 expression was elevated in keratinocytes following skin injury (Long et al., 2018). Recently, Li et al. (Li et al., 2019) found that treatment with MVs overexpressing miR-21 mimic dramatically accelerated wound healing at 24 and 48 h after scratching, but miR-21 inhibitor MVs attenuated the pro-migratory effect. In addition, miR-21 mimic MVs augmented the endotheliocyte angiogenic activity and promoted the fibroblast differentiation compared to miR-21 inhibitor MVs and vector MVs (Li et al., 2019). This is consistent with a previous study (Al-Rawaf et al., 2019). The *in vivo* studies demonstrated that treatment with miR-21 mimic MVs significantly accelerated wound healing compared to treatments with vector MVs and miR-21 inhibitor MVs (Li et al., 2019). PTEN and MAPK/ERK signaling pathways have also been shown to promote cell proliferation and migration (Chen et al., 2018). A in-depth study showed that the treatment with MVs expressing miR-21 mimic significantly reduced the expression of PTEN and RECK, whereas it elevated significantly phosphorERK1/2 (Li et al., 2019). Thus, MV miR-21 may promote fibroblast functions by down-regulating PTEN and RECK in protein level and activating MAPK/ERK signaling cascade, thereby enhancing wound healing.

### miRNA-21 May Exert Anti-Inflammatory Actions and Ameliorate Wound Healing by Regulating the Expression of NF-κB Through PDCD4

Many pathophysiological mechanisms for delayed wound healing have been proposed, one of which, excessive inflammation plays an important role (Russo et al., 2020). The pro-inflammatory cytokines, TNF-α and IL-6 have been reported to aggravate tissue damage (Dinh et al., 2012). Anti-inflammatory cytokine IL-10 facilitates tissue repair by suppressing these inflammatory responses (Obaid et al., 2021). Antibiotics are broadly employed to treat wound infections. However, the effects of antibiotics are poor due to the emergence of multiple-drug resistance bacteria (Armstrong et al., 2017). Recently, platelet-rich plasma (PRP) shows potential as a treatment for wound healing due to its antimicrobial and regenerative properties (Deng et al., 2016). Platelet-rich gel (PRG) is produced from PRP and also has a therapeutic effect on a wound. Importantly, PRG does not induce drug resistance and exhibits synergy with conventional antibiotics (Mercier et al., 2004). Within activated platelets, platelet-derived miRNA-21 was observed to have an antibacterial effect and promote wound healing (Etulain 2018; Nagalla et al., 2011). Programmed cell death 4 (PDCD4) acts as a tumor suppressor regulated by miRNA-21 and has an inhibitory effect on cell proliferation by blocking protein translation (Ajuyah et al., 2019). Nuclear factor-κB (NF-κB), a complex of p50 and p65 subunits, contributes to inflammation by facilitating the expression of TNF-α and IL-6, and inhibiting the expression of IL-10 (Imran et al., 2020). Su et al. (Su et al., 2017) reported that increased PDCD4 expression could increase NF-κB activity, resulting in an increase of TNF-α. It has also been shown that inhibition of miRNA-21 expression increased PDCD4 expression, induced the activation of NF-κB, ultimately leading to increased synthesis of pro-inflammatory cytokines (Ma et al., 2011). A recent study found that human keratinocytes (HaCaT) cell proliferation was severely impaired by *Staphylococcus aureus* (T. Li et al., 2019). Interestingly, this inhibition was significantly reversed by the addition of extract liquid of platelet-rich gel (EPG) (Li et al., 2019). Furthermore, PDCD4, IL-6, and TNF-α were upregulated by *Staphylococcus aureus*, consistent with the activation of the NF-κB signaling pathway, which indicates the perverse healing effect of *Staphylococcus aureus* (Li et al., 2019). After intervention with EPG, the changes of the aforementioned cytokines can be reversed and up-regulation of miRNA-21 was coordinated with the downregulation of PDCD4 and p-p65 (T. Li et al., 2019). Therefore, PRP may perform its anti-inflammatory effect and promote wound healing by targeting the miRNA-21/PDCD4/NF-κB signaling pathway.

### miRNA-21-3p/miRNA-21-5p are Involved in Wound Healing by Regulating Fibroblast Function Through Targeting SPRY1/2

Fibroblasts play critical roles in all stages of wound healing, and normal fibroblasts function is closely related to improved wound healing (Guadarrama-Acevedo et al., 2019). Fibroblasts have been found to be regulated by miRNAs in multiple kinds of diseases, including miRNA-21. Su et al. (Su et al., 2019) reported that miR-494 regulated myocardial infarction by promoting the proliferation and migration of fibroblasts. Madhyastha et al. (Madhyastha et al., 2012) demonstrated that miRNA-21 is involved in fibroblast migration and facilitated diabetic wound healing. Sprouty1/2 (SPRY1/2) is the antagonist of fibroblast growth factor (FGF) pathways and the anti-angiogenic gene. Some scholars have found that the inhibition of SPRY1/2 played an important role in successful wound repair (Liao et al., 2019; Wang et al., 2018). However, whether miRNA-21 promoted wound healing by regulating fibroblast function by reducing SPRY1/2 is unclear. Recently, Wu et al. (Wu et al., 2020) reported that patients with diabetes had a lower expression level of miR-21-3p than those with healthy volunteers. Furthermore, the miR-21-3p expression level was decreased, while SPRY1 expression was increased in fibroblasts with the addition of glucose (Wu et al., 2020). Further study found that miR-21-3p agonist (agomiR-21-3p) enhanced the proliferation of fibroblasts, inhibited apoptosis, inhibited SPRY1 expression, and increased the expression of Collagen III (Col 3), basic fibroblast growth factor, and vascular endothelial growth factor in fibroblasts (Wu et al., 2020). Importantly, the aforementioned effects induced by agomiR-21-3p in fibroblasts were all reversed by the miR-21-3p antagonist (antagomiR-21-3p) (Wu et al., 2020). The studies mentioned above indicate that miR-21-3p enhances fibroblast function. The *in vivo* experimental results further found that the agomiR-21-3p-treated mice showed a high wound closure rate (Wu et al., 2020). In addition, knockout or knockdown of SPRY1 enhanced the proliferation of fibroblasts and inhibited the apoptosis of fibroblasts (Wu et al., 2020). Interestingly, the inhibition of miR-21-3p partially reversed these effects (Wu et al., 2020). This is the same as the research result by Hu et al. (Hu et al., 2018). Overall, miR-21-3p regulated the function of fibroblast by targeting SPRY1 and accelerated diabetic wound healing. Another study demonstrated that miR-21-5p level increased in DFU-derived fibroblast (DFUF) compared to the level in non-diabetic foot fibroblasts (NFFs) and inhibited cell proliferation and migration in DFUFs (Liang et al., 2016). It was revealed that protein SPRY1, integrin associated protein (CD47), signal transducer and activator of transcription 3 (STAT3), S100 calcium-binding protein A10 (S100A10), a reversion-inducing-cysteine-rich protein with kazal motifs (RECK) were direct target gene of miR-21-5p by bioinformatics software analysis (Liang et al., 2016). Another study also showed that miR-21-5p could enhance wound healing by improving angiogenesis and fibroblast function through inhibition of SPRY2 (Wu et al., 2020). Therefore, miR-21-5p may take part in diabetic wound healing by regulating angiogenesis and fibroblast function through targeting its downstream signaling molecules, including SPRY1/2.

### miR-21 Facilitates Wound Healing by Activating Wnt/β-catenin/MMP-7 Signal Pathway

Matrix metalloproteinases (MMPs) are zinc-dependent endopeptidases and have been shown to regulate various aspects of wound healing, including the movement of keratinocytes (Aragona et al., 2017; Puthenedam et al., 2011). Matrix metalloproteinase-7 (MMP-7), also known as matrilysin-1, is a crucial member of the MMPs family and degrades the extracellular matrix (ECM) (Liao et al., 2021). It has been reported that the Wnt/β-catenin pathway is closely associated with the proliferation and migration of keratinocytes (Nusse and Clevers 2017). Xu et al. (Xu et al., 2016) found that blocking of the Wnt/β-catenin signal pathway inhibited the invasion and metastasis of endometriosis tissues by suppressing MMP-7. Zhang et al. (Zhang et al., 2018) reported that overexpression of miR-21 could promote proliferation and differentiation of neural stem cells via targeting the Wnt/β-catenin signaling pathway. However, whether miR-21 is involved in wound healing by regulating Wnt/β-catenin/MMP-7 signal remains unclear. Recently, Lv et al. (Lv et al., 2020) demonstrated that miR-21-5p overexpression could promote the migration of HaCaT cells and increase the expression of Wnt4, β-catenin, and MMP-7. Further study found that the addition of Wnt signaling inhibitor ICG001 significantly reversed the above effects of miR-21-5p overexpression (Lv et al., 2020). Furthermore, using *in vivo* experiments authors discovered that the control group showed around 91, 58 and 44% unhealed wounds on days 5, 10, 15 post-operation (Lv et al., 2020). Importantly, treatment with miR-21-5p resulted in a significantly low unclosed rate of diabetic wounds compared with the control group and this effect could be further enhanced by the combination of human adipose stem cell-derived exosomes (hASC-exos) together with miR-21 (Lv et al., 2020). Therefore, miR-21 may promote proliferation and migration of keratinocytes and increase collagen remodeling via Wnt/β-catenin/MMP-7 pathway, accelerating diabetic wound healing. In summary, the combination of ASC-exos together with miR-21 provides a strategy for diabetic wound healing.

### miR-21 Promotes Wound Healing or Regulates Keloid Relapse Through Inhibition of PTEN that Activated PI3K/Akt Signaling Pathway

The activated the PI3K/Akt pathway not only upregulates the expression of VEGF, but also promotes cell proliferation, migration, angiogenesis, and collagen synthesis, and stimulates wound healing (Wei et al., 2020). The phosphatase and tensin homolog (PTEN) is a dual phosphatase and is able to antagonize the activity of the phosphatidylinositol-4,5-bisphosphate 3-kinase (PI3K) by converting PI(3,4,5)P3 to PI(4,5)P2, which plays an important role in the phosphorylation of Akt, achieving negative regulating of the Akt/PI3K signaling pathway (Wu et al., 2019). Previous studies have demonstrated miR-21 could directly regulate PTEN expression in a variety of cancer cells. Liu et al. (Liu et al., 2019) reported that the suppression of miR-21 promoted ovarian cancer cell apoptosis and reduced ovarian cancer cell proliferation by inhibiting PI3K/Akt activity through targeting PTEN. Zhang et al. (Zhang X et al., 2020) also revealed that miR-21 upregulated the expression levels of PTEN and decreased phosphorylated Akt, which inhibited the proliferation of Wilms’ tumor cells. A recent study showed that miR-21 promoted ROS production through NOX2 regulation by the PI3K pathway in macrophages, which influenced the wound healing process (Liechty et al., 2020). Additional studies have also demonstrated that miR-21 enhanced the migration and proliferation of the HaCaT cells and inhibited inflammation through PI3K/Akt signaling pathway, accelerating the wound healing process (Liu et al., 2021; Wang et al., 2021; Yang et al., 2020). However, the specific mechanism by which miR-21 regulates the PI3K/Akt pathway remains poorly understood. Recently, a study has shown that dendritic cells (DCs) triggered the proliferation of cells by secreting factors and then accelerated wound healing (Vinish et al., 2016). Han et al. (Han et al., 2017) reported that miR-21 promoted wound healing via increasing DCs. Additionally, miR-21 overexpression evidently inhibited PTEN and increased the secretion of p-Akt/Akt, while miR-21 inhibitor had the opposite effect on PTEN and p-Akt/Akt (Han et al., 2017). Further study found that PTEN knockdown dramatically improved the differentiation of DCs and secretion of p-Akt/Akt, but PI3K/Akt inhibitor LY294002 markedly reversed this effect induced by si-PTEN (Han et al., 2017). The above-mentioned studies show that miR-21 contributes to wound healing by activating PI3K/Akt signaling pathway via inhibition of PTEN.

Keloids are characterized by the over-proliferation of fibroblasts and tend to recur due to the stimulation of fibroblast proliferation and additional collagen synthesis (Li et al., 2021). Postoperative adjuvant electron beam (EB) irradiation is considered an effective method to reduce keloid recurrence (Lin et al., 2020). However, the molecular mechanism for EB inhibition of keloid growth is largely unknown. It is well known that autophagy is closely associated with cell proliferation. LC3B-II is an important marker of it. Meanwhile, the upregulation of autophagy has been found in keloids (Okuno et al., 2018). Additionally, miRNAs are considered as an important regulator of autophagy, including miR-21 (Shen et al., 2021; Zhang HH et al., 2020). A recent study showed that after EB irradiation, the expression of miR-21-5p and p-Akt and LC3B-II were significantly downregulated, while the expression level of PTEN was upregulated in keloid fibroblasts compared with control levels (Yan et al., 2020). Further study demonstrated that the percentage of the wound healed area was dramatically decreased in keloid fibroblasts transfected with the miR-21-5p inhibitor (Yan et al., 2020). Moreover, the expression of p-Akt and LC3B-II decreased while the expression of PTEN increasing in cells transfected with the miR-21-5p inhibitor (Yan et al., 2020). At the same time, down-regulation of miR-21-5p could inhibit the migration and invasion ability of keloid fibroblasts (Yan et al., 2020). Consistent with this, Yan et al. (Yan et al., 2016) also reported that PTEN and p-Akt were shown to be involved in the regulation of miR-21-5p on keloid keratinocytes, which might account for the recurrence of keloids. These findings suggested that miR-21-5p inhibition modulates migration and autophagy via PTEN/Akt signaling in EB-irradiated keloid fibroblasts, preventing local invasion and recurrence.

### miR-21 Improves Wound Healing by Accelerating the Proliferation And Migration of Keratinocytes via the Inhibition of PDCD4 and TIMP3

Programmed cell death 4 (PDCD4) is a common tumor suppressor and has been shown to be closely related to tumor development. A recent study showed knockdown of PDCD4 can promote HaCaT cell proliferation, indicating that PDCD4 serves as an essential regulator of keratinocytes (Wang et al., 2015). Fu et al. (Fu et al., 2017) reported that overexpression of miR-21 could inhibit granulosa cells apoptosis by inhibiting the expression of PDCD4. Tissue inhibitor of metalloproteinase-3 (TIMP3) has been reported to suppress the metastasis of glioma cells and breast cancer cells (Hu et al., 2021; Wei et al., 2021). MMP2 is a downstream gene of TIMP3 and affects keratinocyte migration by degrading the extracellular matrix (Caley et al., 2015; Lazaro et al., 2016). Zhang et al. (Zhang et al., 2018) demonstrated that miR-21 promoted the proliferation, migration, and invasion of cervical cancer cells through inhibiting TIMP3. Hu et al. (Hu et al., 2020) claimed that the abundance of TIMP3 was downregulated during wound healing. However, whether miR-21 is involved in the wound healing by targeting PDCD4 and TIMP3 is still underdetermined. Recently, Wang et al. (Wang et al., 2020) found that miR-21 mimics treatment markedly increased keratinocyte proliferation and miR-21inhibitor treatment resulted in a delay in wound healing. In addition, miR-21 mimics efficiently inhibited the expression level of both PDCD4 and TIMP3, while the expression of MMP2 was promoted (Wang et al., 2020). Another study also showed that miR-21 mimics caused a 38% reduction of TIMP3 expression in HaCaT cells (Yang et al., 2011). In summary, miR-21 accelerates the proliferation and migration of keratinocytes by inhibiting the expression of PDCD4 and TIMP3, thereby significantly improving wound healing.

### miR-21 Inhibits Wound Healing by Suppressing the Expression of Leptin

Chronic wounds, such as venous ulcers (VUs), are typically manifested as delayed union, resulting in severe morbidity and mortality (Tsai et al., 2017). However, the clinic therapeutic options are limited deriving from the lack of understanding of the molecular pathology of wound healing inhibition. It has been reported that increased levels of metalloproteinases in the inflammatory phase of venous ulcers destroy proteins essential for ECM formation, thus inhibiting re-epithelialization, revascularization, and closure (De Angelis et al., 2019). Leptin is a circulating anti-obesity hormone and has an effect on wound healing by enhancing re-epithelialization of the wound (Tadokoro et al., 2015). Another study also showed that would healing delayed in leptin-deficient mice and exogenous administration of leptin restored this delayed wound healing (Frank et al., 2000). Pastar et al. (Pastar et al., 2012) found that miR-21 was up-regulated and the expression level of Leptin was suppressed in VUs compared with control skin. Furthermore, the silence of miR-21 significantly increased the levels of Leptin (Pastar et al., 2012). In addition, the wound edges remained almost at the same initial position after treatment with mimic miR-21, suggesting that miR-21 inhibited epithelialization (Pastar et al., 2012). Similarly, miR-21 had also been found to delay wound healing *in vivo* (Pastar et al., 2012). Importantly, the luciferase reporter assay verified Leptin as a direct target for miR-21 (Pastar et al., 2012). The studies mentioned above showed that overexpression of miR-21 inhibits wound healing by inhibiting Leptin. However, Long et al. (Long et al., 2018) demonstrated that miR-21 overexpression significantly improved wound repair in aged mice. These researches indicate that miR-21 may serve different roles in different kinds of the wound.

### miR-21 Promotes the Process of Keloid Fibrosis via the TGF-β/Smad7-Smad2/3 Pathway

Scar formation is widely regarded as an abnormal wound healing response and its pathogenesis is assumed to occur through the recruitment of myofibroblasts, resulting in excessive deposition of ECM. The differentiation of dermal fibroblasts is the primary source of myofibroblasts and is initiated by the TGF-β signaling pathway. TGF-β phosphorylates various Smad family proteins by activating serine/threonine kinase receptor complexes. Phosphorylated Smad2/3 (p-Smad2/3) levels have been proposed as a positive prognostic marker in myofibroblast differentiation. Smad7 belongs to a member of Smad family proteins and is an antagonist of the TGF-β signaling pathway. Additionally, Smad7 inhibits the phosphorylation of Smad2 and Smad3 (Abarca-Zabalia et al., 2020). A recent study found that miR-21-5p contributed to TGF-β inhibition and was important to anti-myofibroblast differentiation in the TGF-β induced human dermal fibroblast, which prevented scar formation during wound healing (Zhang et al., 2021). Fang et al. (Fang et al., 2016) showed that the expression of miR-21 was significantly upregulated in keloids and keloid fibroblasts. Further study demonstrated that miR-21 mimics increased the proliferation rate of fibroblasts and upregulated the expression of TGF-β, P-Smad2 and P-Smad3, while downregulating the expression of Smad7 protein (Wu et al., 2019). However, the miR-21 inhibitor exerted opposite effects (Wu et al., 2019). In addition, Smad7 knockdown could also promote the expression of TGF-β, p-Smad2 and p-Smad3 as well as collagen (Wu et al., 2019). Based on all these results, miR-21 may participate in the process of keloid fibrosis via the TGF-β/Smad7-Smad2/3 pathway, which is consistent with the results deriving from the previous studies (Li et al., 2016).

### miR-21 Involves in the Process of Wound Healing by Regulating the Angiogenic and Inflammatory Pathways

It has been reported that miRNAs regulate wound inflammation by targeting specific key cytokines and related factors. TNF-α, IL-10, and macrophage chemoattractant protein (MCP-1) are known to be involved in the occurrence and development of inflammation. Zhang et al. (Zhang Y et al., 2020) demonstrated that miR-125b inhibited the expression of TNF-α by binding to its 3′-UTR. In addition, IL-10 can be directly regulated by several microRNA, including miR-4661, miR-27, and miR-98 (Quinn and O’Neil 2014). Kawano et al. (Kawano and Nakamachi 2011) showed that miR-124a was directly involved in the post-transcriptional silencing of MCP-1 by targeting MCP-1 and suppressing its expression. As for miR-21, Das et al. (Das et al., 2019) demonstrated that a collagen-based wound-care dressing could regulate the wound macrophage function and thus modify wound inflammation outcomes by interacting with the miR-21-PDCD4-IL-10 pathways.

**FIGURE 1:**
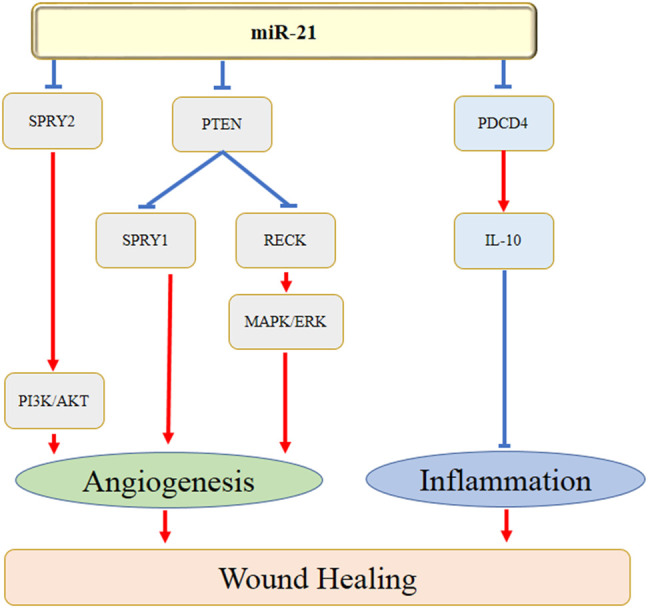
MiR-21 regulates the angiogenesis and inflammation during wound healing.

miRNAs are also found to regulate several aspects of angiogenesis. The homeobox gene GAX and ETS-1, one of the important angiogenesis-related transcription factors, play crucial roles in angiogenesis. It has been reported that miR-130a promoted endothelial cell proliferation by targeting GAX, which induced the process of angiogenesis (An et al., 2017). Furthermore, Chan et al. (Chan et al., 2011) showed that miR-200b knockdown significantly increased angiogenesis by promoting cell migration. The authors further found that suppression of ETS-1 inhibited miR-200b-depended angiogenesis. There are several studies have indicated the roles of miR-21 in treating wound healing by enhancing angiogenesis. Li et al. (Q. Li et al., 2019) revealed that keratinocyte-derived microvesicle miR-21 significantly accelerated skin wound healing by facilitating the process of angiogenesis. The underlying mechanisms were speculated with the down-regulation of PTEN and RECK expression and the activation of MAPK/ERK signaling cascade. A more previous study also demonstrated that exosomes-derived miR-21-3p promoted cutaneous wound healing by the acceleration of angiogenesis, which might be correlated with the inhibition of the level of PTEN and sprouty homolog 1 (SPRY1) (Hu et al., 2018). Figure 1 showed the action of miR-21 in wound healing by regulating the angiogenesis and inflammation.

Akt/NF-kB is one of the well-known pathways to improve angiogenesis. It was suggested that static magnetic field exosomes derived from bone mesenchymal stem cells enhanced wound healing by improving angiogenesis, while the upregulation of miR-21-5p, inhibition of SPRY2 (the target gene for miR-21-5p), and activation of the PI3K/AKT were considered to be the potential mechanisms (Wu et al., 2020).

VEGF plays an important role in inducing neovascularization during the wound healing process through the promotion of the growth, migration, and viability of the endothelial cells. Liu et al. (Liu et al., 2021) reported that adipose-derived stem cells exerted a therapeutic potential in cutaneous wound healing by promoting the growth of dermal fibroblasts and extracellular matrix, which might partially mediated by increasing miR-21 expression and down-regulating its direct target PTEN and MMP1. A previous study indicated that a modified collagen gel resolved wound inflammation and improved angiogenesis by inhibiting miR-21 expression and JNK pathway as well as elevating pro-angiogenic VEGF production (Das et al., 2019). Similarly, Zhang et al. (Zhang et al., 2019) also demonstrated that the promotion of wound healing might be associated with the repression of miR-21 and the up-regulation of VEGF expression. Based on the above evidence, miR-21 exhibited a crucial role in wound healing might partly due to its effect on regulating VEGF expression and the interaction of the related pathways.

Taken together, miR-21 might involve in the process of wound healing by regulating the angiogenic and inflammatory genes and pathways, e.g. PDCD4, PTEN, RECK, SPRY1, SPRY2, MAPK/ERK, and PI3K/AKT (Figure 1).

**FIGURE 2:**
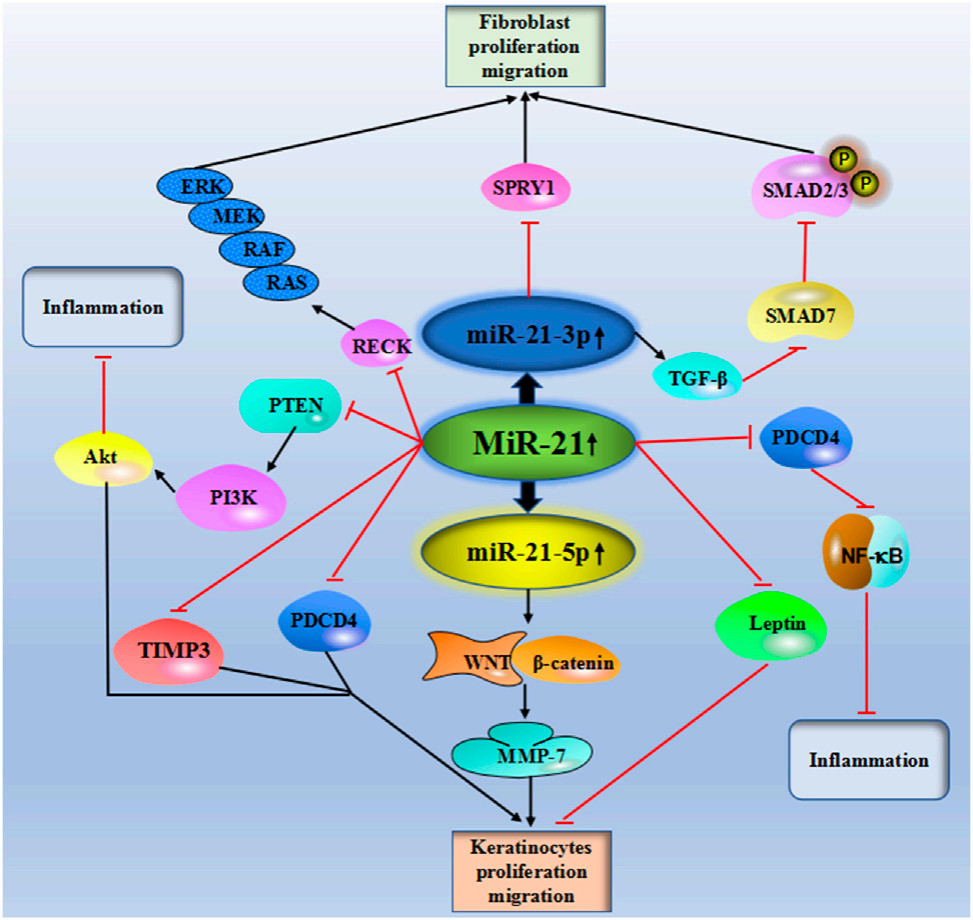
Schematic diagram of the central roles of miR-21 in skin wound healing.

Table 1 and Figure 2 showed the current knowledge of miR-21 in wound healing.

**TABLE 1:** Current knowledge of miR-21 in wound healing.

Study	MiRNAs	Target genes and pathways	Functions of miR-21
Q. Li et al. (2019)	miR-21	PTEN, RECK and MAPK/ERK	Promoting fibroblast functions
T. Li et al. (2019)	miR-21	PDCD4/NF-κB	Inhibiting inflammation
L. Liang et al. (2016), Y. Hu et al. (2018), Y. Wu et al. (2020), and D. Wu et al. (2020)	miRNA-21-3p/miRNA-21-5p	SPRY1/2	Improving angiogenesis and fibroblast function
Q. Lv et al. (2020)	miR-21	Wnt/β-catenin/MMP-7	Promoting proliferation and migration of keratinocytes and increasing collagen remodeling
Z. Han et al. (2017)	miR-21	PTEN/PI3K/Akt	Promoting the migration of keratinocytes and inhibiting inflammation
L. Yan et al. (2016) and L. Yan et al. (2020)	miR-21-5p	PTEN/Akt	Promoting the migration and invasion ability of keloid fibroblasts
X. Yang et al. (2011) and SY. Wang et al. (2020)	miR-21	PDCD4 and TIMP3	Accelerating the proliferation and migration of keratinocytes
Pastar et al. (2012)	miR-21	Leptin	Enhancing re-epithelialization of the wound
S. Fang et al. (2016), G. Li et al. (2016), J. Wu et al. (2019), and Y. Zhang et al. (2021)	miR-21	TGF-β/Smad7-Smad2/3	Increasing the proliferation rate of fibroblasts

## Conclusion and Perspectives

The present review demonstrates that miR-21 may serve as a potential therapeutic target owing to its essential role in wound healing. miR-21 targets the different proteins or signaling pathways, constituting a complex network that promotes or delays wound healing. Currently, miRNA nanocarrier systems have been developed, supporting the feasibility clinical feasibility of such miR-21-based therapy. Though the effects of miR-21 on wound healing have been preliminarily elucidated, the functions of miR-21 in different types of wound repair remain to be further investigations.
